# Auer Rod-Like Inclusions in Reactive Plasma Cells in a Case of Acute Myeloid Leukemia

**DOI:** 10.4274/tjh.2015.0399

**Published:** 2016-12-01

**Authors:** Sarita Pradhan

**Affiliations:** 1 Institute of Medical Sciences and Sum Hospital, Laboratory of Hematology, Bhubaneswar, India

**Keywords:** Auer rods, Acute myeloid leukemia, Plasma cells

A 61-year-old female presented with decreasing hemoglobin for the past 6 months. She had a history of multiple transfusions in the recent past. Laboratory investigations showed hemoglobin of 8.6 g/dL, total blood leukocyte count of 1.13x10^9^/L, and platelets of 80x10^9^/L with the presence of occasional circulating blasts. Bone marrow examination revealed the presence of 63% myeloblasts with prominent Auer rods and mild reactive plasmacytosis (6%). Some of the plasma cells showed Auer rod-like thin slender inclusions (Figures 1, 2, and 3). She was diagnosed with acute myeloid leukemia. Serum protein electrophoresis was done, which showed a normal pattern.

Presence of Auer rod-like inclusions has been described in rare cases of multiple myeloma [1,2], but Auer rod-like inclusions in reactive plasma cells in a case of acute myeloid leukemia have not been reported in the literature. The reported patients either had IgA kappa myeloma or IgG myeloma. Rare cases of Auer rod-like inclusions in aplastic anemia have been reported [3]. However, the exact nature of these inclusions needs to be studied further.

Conflict of Interest: The author of this paper has no conflicts of interest, including specific financial interests, relationships, and/or affiliations relevant to the subject matter or materials included.

## Figures and Tables

**Figure 1:**
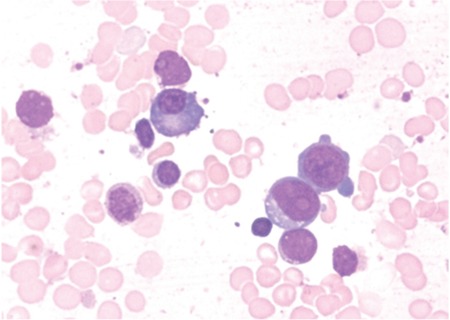
Myeloblasts and plasma cells containing Auer rod-like inclusions.

**Figure 2:**
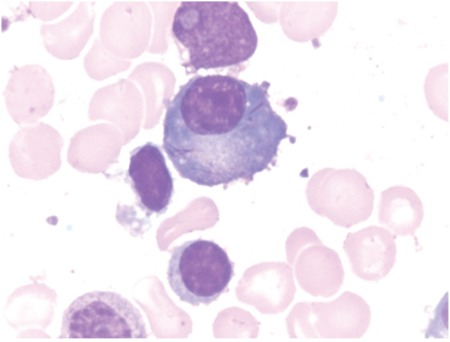
Plasma cell showing Auer rod-like inclusions.

**Figure 3:**
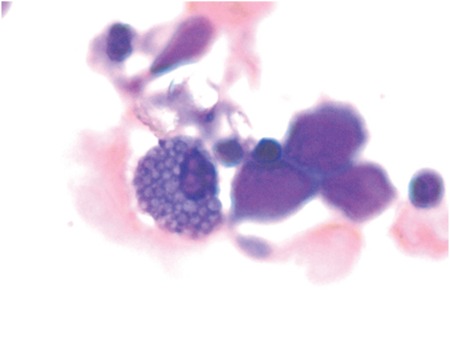
A Mott cell.
